# AutoFactory Dataset to Support AI in Manufacturing Systems

**DOI:** 10.1016/j.dib.2025.111938

**Published:** 2025-07-31

**Authors:** Abderrahmane Boudribila, Abdelouahed Tajer, Zakaria Boulghasoul

**Affiliations:** a Systems Engineering and Applications Laboratory, Cadi Ayyad University, ENSA, BP 2390, Marrakech, 40000, Marrakech-Safi, Morocco; b LAMIGEP, Moroccan School of Engineering Sciences, Avenue Hassan II, Marrakech, 40000, Marrakech-Safi, Morocco

**Keywords:** Artificial intelligence, Named entity recognition, Manufacturing systems, Dataset development, Code automation, Large language models

## Abstract

Automating code generation in manufacturing systems requires Artificial Intelligence (AI) models capable of interpreting textual requirement specifications. One of the main challenges is the absence of publicly available, domain-specific datasets suitable for training such models. This article presents AutoFactory, an open-source dataset that includes manually written and LLM-augmented requirement specifications, annotated by domain experts for Named Entity Recognition (NER) tasks using the BIO format.

AutoFactory enables the extraction and classification of key industrial components, including actuators, pre-actuators, sensors, effectors, and others. These elements correspond to real-world input and output signals in manufacturing systems. The dataset is grounded in realistic scenarios built with Factory I/O and includes 3D scenes, tag tables, and screenshots captured from multiple camera perspectives. To enhance linguistic diversity while preserving semantic intent, all specifications were expanded using large language models and evaluated through semantic similarity analysis (average cosine similarity above 0.92), complemented by manual validation to ensure consistency.

To facilitate the annotation process, a custom labeling tool was developed. It runs locally to preserve data privacy and provides a user-friendly interface. The tool is adaptable to a wide range of NER tasks and allows researchers to customize labels for different domains by adding or modifying tags.

To assess the practical value of the dataset, transformer-based models such as BERT and DistilBERT were fine-tuned on the annotated requirement specifications. The highest-performing model reached an F1-score of 0.95, confirming the dataset’s ability to support accurate identification of key components in manufacturing systems.

This combination of human expertise and AI-based augmentation provides a robust foundation for training AI systems to interpret manufacturing behaviors from text. AutoFactory contributes to ongoing research efforts aimed at enabling intelligent systems to assist in generating control code from requirement specifications, reducing reliance on manual programming in industrial automation.

Specifications Table

**Table:** 

Subject	Engineering & Materials science
Specific subject area	*Artificial intelligence for automated code generation in manufacturing systems*
Type of data	Table, Processed, Annotated
Data collection	*The dataset was created by manually drafting baseline requirement specifications for manufacturing systems. These specifications were expanded into multiple variations using large language models (ChatGPT Pro, Claude Pro, and Mistral Pro). After expert validation, each specification was formally annotated using a custom labeling tool with BIO tags (Begin, Inside, Outside) for Named Entity Recognition (NER).*
Data source location	*Systems Engineering and Applications Laboratory, Cadi Ayyad University, ENSA, BP 2390, Marrakech, 40,000, Marrakech-Safi, Morocco.* *The dataset is publicly available on Hugging Face:*https://huggingface.co/datasets/boudribila/AutoFactory*DOI:**10.57967/hf/5011*
Data accessibility	Repository name: Hugging Face Data identification number: *10.57967/hf/5011* Direct URL to data: https://huggingface.co/datasets/boudribila/AutoFactory The dataset can be freely accessed and downloaded directly via the provided URL without restrictions.
Related research article	*Boudribila, A., Chadi, M.A., Tajer, A. and Boulghasoul, Z., 2023, July. Large language models and adversarial reinforcement learning to automate PLCs programming: A preliminary investigation. In 2023 9th International Conference on Control, Decision and Information Technologies (CoDIT) (pp. 650–655). IEEE.*

## 1 Value of the Data

• The dataset provides clearly annotated manufacturing requirements specifications, addressing the absence of publicly available datasets for industrial automation. This allows researchers to train AI models capable of interpreting manufacturing instructions and supports research into methods for generating control logic, with the potential to reduce human intervention.
• Researchers can reuse these data to build models that identify critical components, such as actuators, pre-actuators, sensors, and effectors, enabling more effective transformation of textual specifications into executable control programs.
• Annotations use the standard BIO tagging format (Begin, Inside, Outside), ensuring compatibility with widely adopted NLP and machine learning frameworks, and simplifying integration into existing pipelines.
• The dataset includes scenes of 3D real manufacturing systems built using Factory I/O, a simulation platform for industrial automation. Each system is accompanied by tag tables and screenshots from multiple camera angles, as well as the corresponding 3D scene file, the baseline requirement specification, its augmented versions, and a similarity analysis using sentence embeddings to ensure both linguistic diversity and semantic consistency.
• The dataset serves as a reliable benchmark to evaluate and compare AI methodologies. It includes realistic manufacturing examples originally written or reviewed by domain experts then reformulated using large language models (ChatGPT Pro, Claude Pro, Mistral Pro) to introduce controlled linguistic diversity while preserving technical meaning.
• All annotations were reviewed and validated by human experts during the labeling process, ensuring high accuracy and reliability. This makes the dataset suitable for both academic research and industrial applications.
• The dataset is hosted publicly on Hugging Face with a permanent DOI, ensuring long-term access for researchers, developers and educators.

## 2 Background

Automated manufacturing systems rely on Programmable Logic Controllers (PLCs) to control and monitor industrial processes [1]. In practice, engineers use two main strategies to program PLCs. The first is a heuristic approach [2,3], where engineers read the requirement specifications and directly write the control program based on their experience and intuition. The second is a formal approach [2], where engineers first create mathematical models of the system behavior and its operational constraints before generating the program. Although both methods are well established, they are time-consuming, prone to human error, and require strong technical expertise.

In our work, we aim to move beyond these traditional methods by using artificial intelligence (AI) to automate the generation of PLC programs directly from textual requirement specifications. However, during a pilot study, we found that no public datasets existed to train AI models for this specific task. To overcome this challenge, we manually wrote realistic requirement specifications based on systems modeled in Factory I/O. We then expanded these specifications using large language models (LLMs), rewriting them with different words while keeping the original meaning. Each version was verified by human experts to ensure accuracy, and similarity metrics were used to confirm that the reformulations preserved the original intent.

This dataset complements our previous research [4] and provides a structured, domain-specific resource for advancing AI-based automation in manufacturing.

## 3 Data Description

In automated manufacturing systems, engineers, students, and system designers often describe the intended behavior of machines using natural language before writing any control code. These requirement specifications typically indicate what the system should do (e.g., extend a cylinder), what it must avoid (e.g., exceed a safety limit), and which components are involved (e.g., sensors, valves, or actuators). The purpose of the AutoFactory dataset is to enable machines to take such natural language as input, understand its meaning, identify the key components mentioned, and then support the automatic generation of control logic that would normally be written manually by engineers.

To support this goal, AutoFactory [5] provides 2358 manually annotated requirement specifications based on realistic industrial scenarios modeled in Factory I/O. Each specification represents a complete industrial process, including operation sequences, safety conditions and interactions between system components. The dataset consists of 17,134 segmented sentences and a total of 279,830 tokens. Each token is annotated and structured into five columns, following a format adapted from the CoNLL-2003 standard for Named Entity Recognition (NER) tasks:

• **Token text:** the word or punctuation (e.g., Cylinder, Controlled)
• **POS tag:** the part-of-speech label (e.g., noun [NN], verb [VB], determiner [DT])
• **POS index:** the numeric code for the POS tag (e.g., NN = 10)
• **NER label:** the named entity tag identifying the component type (e.g., ACTUATOR, SENSOR)
• **NER index:** the numeric code for the NER label (e.g., B-ACTUATOR = 1)

The NER labels follow the BIO tagging scheme:

• **B (Beginning):** marks the first word of an entity (e.g., B-ACTUATOR)
• **I (Inside):** marks the continuation of the same entity (e.g., I-ACTUATOR)
• **O (Outside):** indicates tokens that do not belong to any labeled entity

This format allows the model to recognize multi-word technical components. For example, in the phrase “double-acting cylinder,” “double-acting” is labeled as B-ACTUATOR and “cylinder” as I-ACTUATOR, forming a single entity.

Table 1 shows an example of a token-level annotation from a requirement specification in the dataset.

**Table 1:** Example of Token-Level Annotation in the AutoFactory Dataset.

Token	POS	POS_numeric	NER Label	NER_numeric
The	DT	5	O	0
system	NN	10	O	0
consists	VBZ	25	O	0
of	IN	7	O	0
a	DT	5	O	0
conveyor	NN	10	B-EFFECTOR	7
belt	NN	10	I-EFFECTOR	8
driven	VBN	23	O	0
by	IN	7	O	0
an	DT	5	O	0
electric	JJ	8	B-ACTUATOR	1
motor	NN	10	I-ACTUATOR	2

Each requirement specification consists of multiple sentences, with empty lines marking sentence boundaries. All annotations were created and reviewed manually by domain experts to ensure accuracy and consistency. The dataset includes both POS and NER labels, each mapped to a numeric index for compatibility with standard machine learning frameworks. The POS tagging helps models understand grammatical structure, and NER labeling helps identify technical components, such as ACTUATOR (e.g., cylinders, motors), PREACTUATOR (e.g., valves, contactors), SENSOR (e.g., limit switches, detectors), EFFECTOR (e.g., conveyor belt, lights), and OTHER (tokens not linked to physical control elements).

**Fig. 1:**
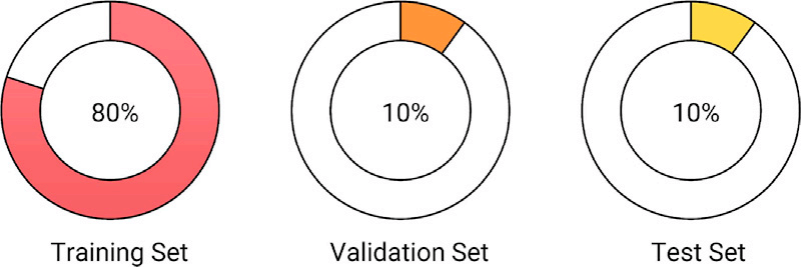
Dataset Split – Training, Validation, and Test Sets.

The dataset is provided in both Excel (.xlsx) and CSV (.csv) formats, encoded in UTF-8 to ensure compatibility with standard natural language processing and machine learning frameworks. It follows a conventional 80–10–10 split: 80 % of the data is allocated to the training set, 10 % to the validation set, and 10 % to the test set. Fig. 1 illustrates this partitioning.

• Training Set (80 %): used to train the models
• Validation Set (10 %): used for hyperparameter tuning
• Test Set (10 %): used for final model evaluation

Table 2 summarizes the dataset’s structure and semantic annotations. It includes 2358 requirement specifications, segmented into 17,134 sentences and comprising 279,830 tokens. Among these tokens, 7.09 % are labeled as ACTUATOR, 2.11 % as PRE-ACTUATOR, 9.18 % as SENSOR, and 9.52 % as EFFECTOR. The remaining 72.09 % of tokens are labeled as OTHER, indicating they do not refer to system components but contribute to grammatical structure and contextual clarity. These proportions reflect the natural language composition of industrial specifications, where functional terms are embedded within descriptive text.

**Table 2:** Summary Statistics of Specifications, Tokens, and Named Entities in AutoFactory.

Dataset	Specifications	Sentences	Tokens	Actuators	Pre-Actuators	Sensors	Effectors	Others
Training	1886	13,686	223,003	15,838	4645	20,492	21,133	160,895
Validation	236	1728	28,759	2030	684	2634	2785	20,626
Test	236	1720	28,068	1983	584	2569	2722	20,210
**Total**	**2358**	**17,134**	**279,830**	**19,851**	**5913**	**25,695**	**26,640**	**201,731**

**Fig. 2:**
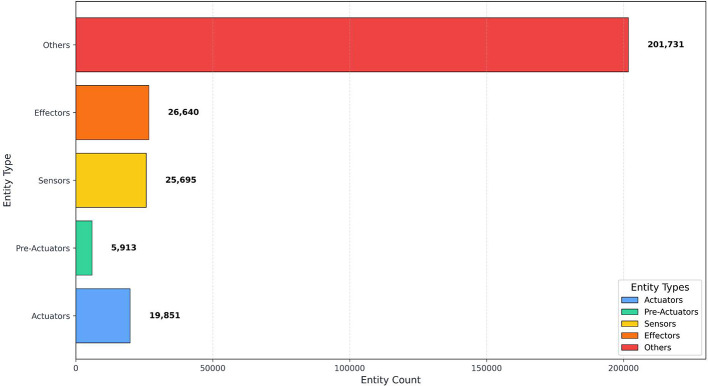
Distribution of Labeled Entities in the AutoFactory Dataset.

To further illustrate the component distribution, Fig. 2 visualizes the relative frequency of each label category. Actuator and sensor mentions are most prevalent, consistent with their key role in control system logic. Pre-actuators and effectors appear less frequently but remain essential for modeling system transitions.

The AutoFactory dataset is designed not only for engineers in industrial automation but also for researchers, developers, and students working in natural language processing and AI. It serves as a high-quality benchmark for tasks such as named entity recognition, information extraction, and the automatic generation of control logic from textual specifications. The dataset offers a structured, modular format that is both accessible and extensible, making it suitable for academic research and industrial applications alike.

Despite significant progress in Named Entity Recognition (NER), existing datasets have not addressed the specific challenges of understanding requirement specifications in industrial automation. Most NER corpora, such as CoNLL-2003 [6], FabNER [7], and MS-NERC [8], focus on general-purpose or scientific language, but none are structured to represent how engineers describe the behavior of components in manufacturing systems. AutoFactory fills this gap by providing fine-grained annotations aligned with how control engineers express system behavior. Each annotated specification represents a full operational scenario modeled in Factory I/O, covering input/output components, event sequences, and safety constraints. This alignment with executable industrial scenarios enables researchers to train models that not only recognize entities, but also learn to infer control logic structures from language.

As shown in Table 3, AutoFactory is currently the only open-access dataset that provides linguistically and semantically annotated manufacturing requirements grounded in system behavior modeled using Factory I/O. This contributes to developing AI models that can interpret requirement specifications and support the generation of control code in industrial automation.

**Table 3:** Comparison of AutoFactory with existing domain-specific and general-purpose NER datasets.

Feature	AutoFactory	CoNLL-2003	FabNER	MS-NERC
Domain	Requirement specifications for manufacturing systems (industrial automation)	General news articles (Reuters Corpus)	Scientific abstracts in manufacturing research	Aerospace manufacturing specifications
Language	English	English and German	English	Chinese
NER Tags	5 categories, including (ACTUATOR, PRE-ACTUATOR, SENSOR, EFFECTOR, OTHER)	4 categories, including (PERSON, LOCATION, ORGANIZATION, MISCELLANEOUS)	12 categories, including (MATERIAL, MANUFACTURING PROCESS, MACHINE, APPLICATION, ENGINEERING FEATURES, etc.)	16 categories, including (PART, PART_ID, MATERIAL, ATTRIBUTE, etc.)
Tag Format	BIO (Begin-Inside-Outside)	BIO (Begin-Inside-Outside)	BIOES (Begin-Inside-Outside-End-Single)	BIOE (Begin-Inside-Outside-End)
Data Source	Manually written requirement specifications expanded by large language models and annotated by experts	News articles from Reuters (English) and Frankfurter Rundschau (German), manually annotated for named entities	Scientific abstracts from manufacturing journals and textbooks, manually labeled for technical terms	Chinese aerospace manufacturing specifications, annotated manually by domain experts
NER Purpose	Supports AI systems that generate control code for industrial production systems	Supports AI systems for testing and comparing named entity recognition methods on news articles	Supports AI systems that extract scientific concepts from manufacturing process research	Supports AI systems that identify technical components in aerospace manufacturing specifications
Use Case	Training AI models to extract components and generate control programs for industrial automation	Evaluating and benchmarking general-purpose Named Entity Recognition (NER) systems	Extracting technical knowledge from manufacturing research for information retrieval and topic modeling	Identifying technical entities to support information extraction in aerospace manufacturing specifications
Availability	Open access	Open access	Open access	Restricted access (on request)

**Fig. 3:**
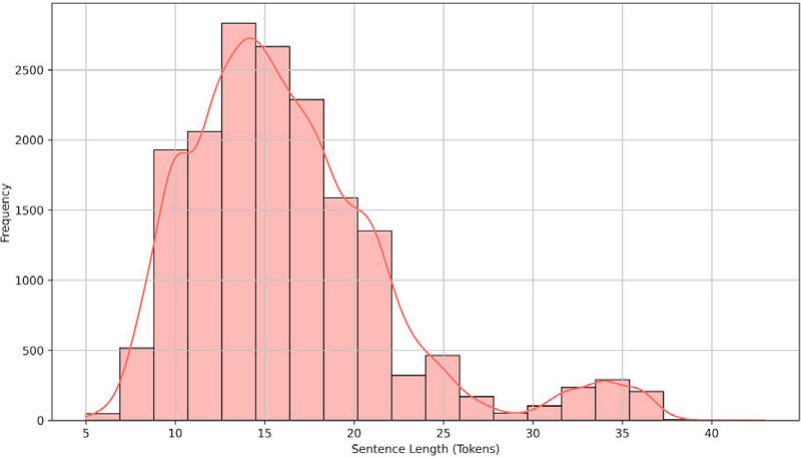
Histogram of Sentence Lengths (in Tokens) Across the AutoFactory Dataset.

To evaluate the structural complexity of the dataset, we analyzed sentence length across all annotated specifications. Sentence length provides a basic yet informative indicator of linguistic variation and syntactic richness. As shown in Fig. 3, lengths range from 5 to over 40 tokens, with a concentration around 14 to 16 tokens. While many sentences are relatively short, the distribution includes a substantial number of longer sequences, reflecting more detailed or compound requirement descriptions. This variation supports the development of models that can process both simple and complex expressions of control behavior, and it demonstrates that the dataset captures the range of natural language found in real industrial contexts.

In addition to sentence length, we examined the diversity of part-of-speech (POS) tag sequences to assess structural variability beyond surface-level token counts. Table 4 summarizes the number of unique POS patterns identified across the three systems and the full dataset. AutoFactory contains 4778 distinct POS tag sequences across 17,134 sentences, corresponding to a diversity ratio of 27.89 %. This metric reflects the proportion of unique syntactic structures relative to total sentences. System 1 and System 3 show particularly high diversity, with ratios exceeding 28 %, indicating greater variability in how requirements are phrased. These findings confirm that the dataset includes a wide range of grammatical constructions, which can help AI models learn robust generalizations across different linguistic forms of technical specifications.

**Table 4:** POS Tag Sequence Diversity Across Individual Systems and the Full Dataset.

Metric	System 1	System 2	System 3	AutoFactory (All)
Total Sentences	5335	5168	6602	17,134
Unique POS Sequences	1791	1108	1883	4778
Diversity Ratio (%)	33.57 %	21.44 %	28.52 %	27.89 %

To further evaluate lexical variability, we measured n-gram diversity and entropy across the dataset. Table 5 reports the number of unique bigrams and trigrams, as well as their corresponding entropy scores. The full AutoFactory dataset contains 8078 distinct trigrams and 3300 bigrams, with trigram entropy reaching 10.56. These values indicate a high level of lexical diversity and reduced repetition across specifications. Among individual systems, System 3 exhibits the highest bigram and trigram entropy, consistent with its longer and more structurally varied sentences. High entropy values suggest that the dataset avoids rigid or repetitive phrasing, offering a broader lexical space for training models to generalize across different ways of expressing functional requirements.

**Table 5:** N-gram Diversity and Entropy Across Individual Systems and the Full Dataset.

N-gram Metric	System 1	System 2	System 3	AutoFactory (All)
Unique Bigrams	1734	1086	1620	3300
Bigram Entropy	8.4113	7.8143	8.5357	9.1402
Unique Trigrams	3883	2144	3527	8078
Trigram Entropy	9.7597	8.8655	9.5719	10.5591

To assess lexical diversity, we computed the type-token ratio (TTR) and distinct-n metrics, which measure vocabulary variety and repetition. As shown in Table 6, AutoFactory contains 279,830 tokens and 629 unique token types, resulting in a TTR of 0.00225. These values are consistent with the dataset’s current structure, which includes only 3 out of the 21 Factory I/O systems. Each reformulated specification describes the same system using a consistent set of component names. As a result, the vocabulary reflects technical consistency rather than linguistic redundancy. The distinct-1 and distinct-2 scores further confirm that the dataset maintains meaningful variation in phrasing across reformulations. As additional Factory I/O systems are incorporated, we expect greater lexical diversity due to the introduction of new components and system behaviors.

**Table 6:** Token Diversity Metrics Across Individual Systems and the Full Dataset.

Metric	System 1	System 2	System 3	AutoFactory (All)
Total Tokens	81,784	80,428	117,198	279,830
Unique Tokens	398	308	368	629
TTR	0.00487	0.00383	0.00314	0.00225
Distinct-1	0.00487	0.00383	0.00314	0.00225
Distinct-2	0.02120	0.01350	0.01382	0.01179

To further evaluate linguistic diversity, we measured Self-BLEU scores across the dataset. Self-BLEU quantifies how similar each sentence is to others within the same set, with lower scores indicating higher diversity. As shown in Table 7, the average Self-BLEU score for AutoFactory is 0.9984, with a minimum of 0.9510 and a standard deviation of 0.0045. These high values reflect the dataset’s design, where each set of specifications reformulates the same base scenario using consistent technical components. Despite this structural consistency, LLM-generated reformulations still produce varied sentence constructions, preserving diversity in expression. As AutoFactory expands to include more Factory I/O systems, the range of sentence patterns and inter-sentence variability is expected to increase accordingly.

**Table 7:** Self-BLEU Evaluation Across Individual Systems and the Full Dataset.

Statistic	System 1	System 2	System 3	AutoFactory (All)
Average Self-BLEU	0.9970	0.9983	0.9982	0.9984
Minimum	0.9448	0.9551	0.9678	0.9510
Standard Deviation	0.0066	0.0054	0.0043	0.0045

To assess syntactic complexity, we measured the depth of syntactic parse trees for each sentence. A deeper tree generally indicates a more complex sentence structure. As shown in Table 8, the average parse depth in AutoFactory is 22.51, with System 3 having the highest average at 33.36. The depth ranges from 11 to 59, showing that the dataset includes both simple and complex sentence constructions. These variations reflect the natural differences in how requirements can be expressed, even when describing the same system. The inclusion of multiple sentence types supports the development of models that can handle a range of syntactic forms. As more systems are added in future versions, the variety of sentence structures is expected to grow further.

**Table 8:** Syntactic parse tree depth statistics Across Individual Systems and the Full Dataset.

Statistic	System 1	System 2	System 3	AutoFactory (All)
Number of Sentences	899	748	711	2358
Average Depth	15.15	21.04	33.36	22.51
Minimum Depth	11	13	20	11
Maximum Depth	21	38	59	59
Standard Deviation	1.73	3.71	6.58	8.68
25th Percentile	14.0	19.0	28.0	16.0
Median Depth	15.0	21.0	33.0	20.0
75th Percentile	16.0	23.0	37.0	28.0

The AutoFactory dataset combines consistent semantic content with diverse linguistic expressions grounded in real Factory I/O scenarios. Although this initial version includes only 3 out of 21 available systems, it already reflects a wide range of sentence structures, grammatical patterns, and technical components. This variation is not the result of random or superficial reformulation, but reflects the different ways engineers naturally express similar control behaviors. The dataset integrates multiple layers of annotation including part-of-speech tags, named entity labels and syntactic parse depth which support both surface-level and structural language analysis. These features make AutoFactory a valuable resource for developing models that can understand and extract control-relevant information from textual specifications. To further validate this potential, the next section presents baseline experiments for named entity recognition using transformer-based models.

## 4 Experimental Design, Materials and Methods

The AutoFactory dataset was developed to address a pressing limitation in the field of artificial intelligence for industrial automation. While recent advances in automation have transformed manufacturing systems, there remains a lack of publicly available datasets that support the automatic interpretation of requirement specifications and the generation of Programmable Logic Controller (PLC) code. Most existing natural language processing datasets are designed for general-purpose tasks, such as recognizing names, locations, or organizations. These resources are insufficient for industrial applications, which require the identification and interpretation of technical components, including actuators, valves, sensors, and control behaviors. AutoFactory was created to fill this gap by offering a domain-specific, annotated dataset grounded in realistic manufacturing scenarios.

**Fig. 4:**
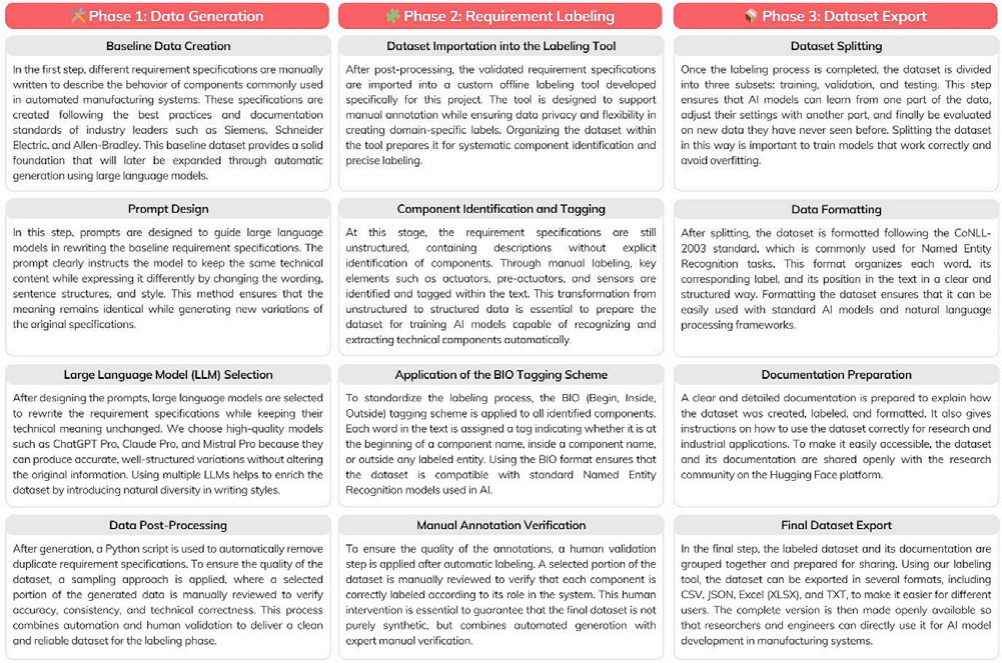
Structured Workflow for the Construction of the AutoFactory Dataset.

The dataset construction followed a structured three-phase methodology: specification generation, component annotation, and dataset preparation. The complete process is illustrated in Fig. 4.

In the first phase, a set of initial requirement specifications was manually written to describe the behavior of three manufacturing systems modeled in Factory I/O. These specifications were then expanded using large language models to produce linguistically diverse variants that preserve the same functional meaning. In the second phase, domain experts manually annotated the specifications using a BIO tagging scheme to identify key industrial components, including actuators, pre-actuators, sensors, and effectors. In the third phase, the annotated data was structured and exported in multiple formats (CSV, XLSX), ensuring compatibility with machine learning tools and facilitating its use for training and evaluating AI models in industrial automation tasks.

## 5 Dataset Construction

To train AI systems that can understand requirement specifications, it is important to have data that reflects how real manufacturing systems are described. However, there are currently no public datasets built for this purpose. Without such data, it becomes difficult to develop models that can detect and classify the main components used in automated systems.

To solve this problem, the AutoFactory dataset was built using a structured approach based on industrial systems modeled in Factory I/O. These virtual systems simulate realistic automation scenarios and were used as a starting point to write clear and complete requirement specifications. Each specification was manually written to describe the behavior of machines in these scenes.

To increase variety in how requirements are expressed, large language models were used to generate different versions of each original specification. These versions were reviewed by domain experts to make sure they kept the same meaning and were technically accurate. Finally, the data was cleaned and organized to remove duplicates and fix any small errors, resulting in a well-structured and high-quality dataset for research in industrial automation.

## 6 Baseline Data Creation

The first step in creating the dataset is to write baseline requirement specifications for components commonly found in automated production systems. These specifications serve as the foundation of the dataset and provide clear and structured descriptions of how different manufacturing components operate. Since there are no publicly available datasets for this purpose, we manually create these specifications, following the best practices and documentation standards used by leading global companies in industrial automation, such as Siemens (Germany), Schneider Electric (France), and Allen-Bradley (USA).

**Fig. 5:**
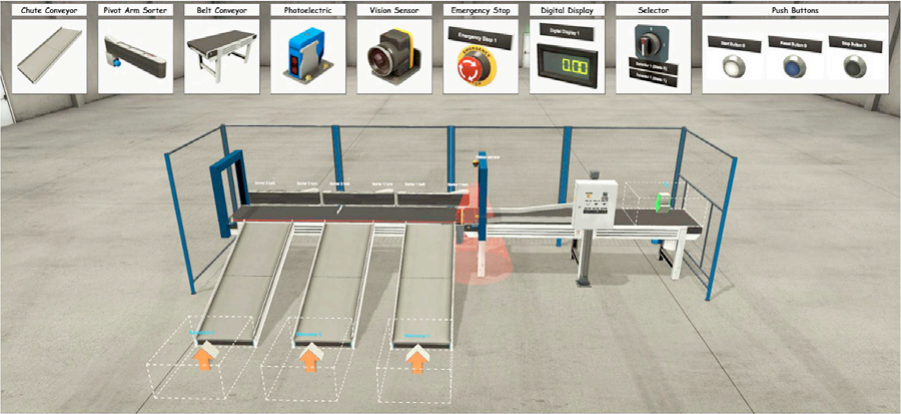
Example of a manufacturing system: Sorting Station composed of multiple components working together.

Automated manufacturing systems are made up of many parts, or components, that work together to complete tasks. For example, a system may have cylinders, conveyor belts, sensors, and motors. Each of these components has its own role, and together they form a complex system (see Fig. 5).

A sorting station, is an example of such a system. It consists of different components, such as conveyors, vision sensors, and sorting mechanisms, that work together to classify and transport items. Each component plays a distinct role in the process, contributing to the overall system functionality.

To capture this, each baseline requirement specification represents the full functional logic of a selected manufacturing system, combining multiple components into a coherent operational flow. Rather than isolating components, the specification explains how actuators (e.g., double-acting cylinders, electric motors), pre-actuators (e.g., contactors, solenoid valves), sensors (e.g., inductive sensors, limit switches), and effectors (e.g., conveyor belts, pop-up sorters) interact to perform specific tasks.

This version of the dataset includes three representative manufacturing systems modeled in Factory I/O: a single conveyor belt transport system, a dual conveyor belt system, and a dual conveyor system with a transfer cylinder. These systems were selected because they contain a wide range of commonly used industrial components, such as start buttons, contactors, electric motors, photoelectric sensors, and double-acting cylinders. Each system reflects realistic operational logic typically implemented in programmable logic controller (PLC)-based environments.

Each requirement specification captures the functional behavior of a complete manufacturing system. The descriptions include triggering conditions, sequences of component activations, and terminal states. This structure ensures that the specifications not only reflect the vocabulary of industrial automation but also convey the relationships between sensors, actuators, pre-actuators, and effectors within each operational cycle. Such modeling is essential for training AI systems to identify entities and understand control logic in natural language.

To illustrate the nature of the specifications, Table 9 provides examples of the baseline manufacturing systems included in the dataset. Each specification offers a coherent narrative describing how the system responds to external inputs and internal conditions to achieve its intended functionality.

**Table 9:** Baseline Requirement Specifications for Selected Factory I/O Systems.

System Name	Requirement Specification
Single Conveyor Belt Transport System	The system consists of a conveyor belt driven by an electric motor. A normally open start button is used to initiate the transport cycle. When the start button is pressed, the start light is turned on and the conveyor motor is activated. A photoelectric exit sensor is installed at the end of the conveyor. When the sensor detects the presence of a box, the conveyor motor stops and the start light turns off. The system waits for the next press of the start button to repeat the cycle.
Dual Conveyor Belt Transport System	The system consists of two conveyor belts, each driven by an electric motor: Conveyor One and Conveyor Two. A start button is used to initiate the cycle. When the start button is pressed, the start light turns on, and Conveyor One is activated. The box moves forward until it reaches Sensor A. Once Sensor A is triggered, Conveyor One stops, and Conveyor Two is activated. The box continues moving until it reaches Sensor B, which stops Conveyor Two. The start light then turns off. The system is now idle and waits for the next press of the start button to repeat the cycle.
Dual Conveyor Belt System with Transfer Cylinder	The system consists of two conveyor belts, each driven by an electric motor and controlled by contactors KM1 and KM2. A double-acting cylinder is used to transfer boxes between the conveyors. The cylinder is controlled by a 5/2-way solenoid valve. A start button is used to initiate the cycle. When the start button is pressed, the start light turns on. At the same time, Conveyor One and Conveyor Two are both activated. The box on Conveyor One moves forward until it reaches the entry sensor. Once the entry sensor is triggered, Conveyor One stops. The double-acting cylinder then extends forward. It continues moving until it activates the front limit switch. When the front limit switch is triggered, the solenoid valve is reversed, and the cylinder retracts. The cylinder moves back until the back limit switch is triggered. Conveyor Two continues running during the entire process. When the exit sensor detects the presence of the box at the end of Conveyor Two, and the cylinder has fully retracted, the system restarts the cycle. Conveyor One is activated again to bring the next box forward.

Each system is also accompanied by its Factory I/O scene file, tag documentation, and multi-angle screenshots to support reproducibility. These baseline specifications serve as the core data from which further linguistic variants are generated in the following stage.

## 7 Prompt Design

To train AI models that can understand requirement specifications, it is important to provide them with a large amount of varied data. In real industrial settings, different engineers often describe the same system in different ways. They may use different words, sentence structures, or levels of detail, depending on their experience and writing style. These differences do not change the meaning but affect how the information is presented. For AI models to perform well, they must be trained on examples that reflect this diversity.

To introduce this variation, large language models (LLMs) are used to rewrite each baseline requirement specification into multiple versions. The goal is to generate linguistically diverse examples while preserving the original technical meaning. LLMs have demonstrated strong capabilities across various domains, including engineering, and can be guided to produce high-quality paraphrases when given clear instructions.

For each baseline requirement specification, the model is asked to produce three distinct formulations. To ensure consistency and accuracy, the generation process is guided by a structured prompt:

"Rewrite the following requirement specification in three different ways. Keep the technical details the same, but use different wording and sentence structures."

This prompt encourages the model to retain the functional content of the specification while varying its surface form. By applying this method, the dataset captures the natural variability in how requirement specifications are written. This enhances the model’s ability to understand technical content expressed in different ways, making it more robust when applied to real-world industrial texts.

## 8 Large Language Model (LLM) Selection

Once the prompts were designed, the next step involved selecting large language models capable of rewriting requirement specifications while preserving their technical meaning. The reliability and usefulness of the dataset depend directly on the ability of these models to produce accurate and readable variants of the original specifications. Not all language models meet these criteria. Some may omit key details, alter the intended logic, or produce output that lacks technical clarity. It is therefore essential to carefully select models that can maintain both syntactic coherence and semantic fidelity.

To address this, we selected three high-performance models: ChatGPT Pro, Claude Pro, and Mistral Pro. These models were chosen for their ability to generate structured and technically accurate language. Unlike basic paraphrasing tools that rely on synonym replacement, these advanced models can restructure entire sentences and vary lexical choices while preserving the core information. This capacity is essential for building a dataset that reflects the natural variability observed in how different engineers describe the same system.

Each model was applied to the same set of baseline specifications to produce multiple reformulations. The outputs were collected and organized as unlabeled text variants to increase linguistic diversity in the dataset. By using multiple models, we reduce the risk of stylistic bias and improve the generalizability of future AI systems trained on this data.

Table 10 presents an example of a baseline requirement specification followed by three rewritten versions generated by the selected LLMs. These examples illustrate how the same technical content can be expressed differently across models while preserving its meaning and structure.

**Table 10:** Reformulated Requirement Specifications Generated by LLMs.

Source	Reformulated Requirement Specification
Original	The system consists of a conveyor belt driven by an electric motor. A normally open start button is used to initiate the transport cycle. When the start button is pressed, the start light is turned on and the conveyor motor is activated. A photoelectric exit sensor is installed at the end of the conveyor. When the sensor detects the presence of a box, the conveyor motor stops and the start light turns off. The system waits for the next press of the start button to repeat the cycle.
**ChatGPT** (Pro Version)	This automated system includes a conveyor belt driven by a motor. A normally open start button is provided to launch the transport cycle. Once pressed, the system powers on the start light and sets the motor in motion to drive the conveyor. A photoelectric presence sensor installed at the discharge point detects incoming boxes. When a box is detected, the motor stops and the start light is turned off. The system then resets and waits for the next cycle to be initiated by pressing the start button.
**Claude** (Pro Version)	In this system, a conveyor belt is driven by an electric motor. The transport cycle is initiated by pressing a normally open start button. Once pressed, the start light is activated and the motor powers the conveyor. At the end of the conveyor, a photoelectric exit sensor monitors for boxes. When a box is detected, the motor is stopped and the start light is turned off. The system then waits for another start button press to begin the next operation.
**Mistral** (Pro Version)	This system uses an electric motor to drive a conveyor belt. The transport process is triggered by a normally open push button start. Pressing this button switches on the start light and activates the conveyor motor. At the end of the belt, a photoelectric sensor is installed to monitor for the presence of boxes. When the sensor detects a box, the motor is stopped and the start light is turned off. The cycle is then paused until the operator presses the start button again.

To make sure that the requirement specifications generated by large language models (LLMs) keep the same meaning as the original ones, we performed a similarity check. This is an important step, because while the goal is to create different versions using new words and sentence structures, the meaning of the requirement must not change. In industrial systems, even small changes in meaning can lead to incorrect behavior, so it is essential to verify that the information is still correct.

We used cosine similarity to compare each rewritten specification to the original. This method works by converting sentences into numbers (called embeddings) and then measuring how close they are. A high score means that the two sentences are very similar in meaning, even if they are written differently.

**Fig. 6:**
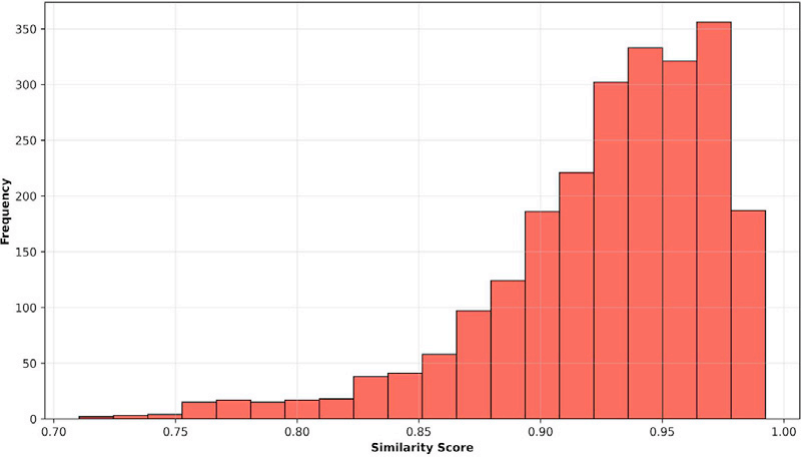
Distribution of similarity scores across all rewritten requirement specifications.

The results show that the rewritten specifications are very close to the original ones. Across the entire dataset, the average similarity score was 0.9275, and the median score was 0.9389. Even the lowest score was 0.7105, which means that no rewritten version introduced major changes. These findings are summarized in Table 11, and the distribution of similarity scores is visualized in Fig. 6.

**Table 11:** Semantic Similarity Metrics between Original and Augmented Specifications.

Metric	System 1	System 2	System 3	AutoFactory (All)
Average ± Std. Dev	0.9172 ± 0.0566	0.9546 ± 0.0295	0.9107 ± 0.0337	0.9275 ± 0.0399
Min / Max Similarity	0.7105 / 0.9879	0.8461 / 0.9924	0.7951 / 0.9849	0.7105 / 0.9924
Median Similarity	0.9363	0.9646	0.9158	0.9389
25th / 75th Percentiles	0.8959 / 0.9576	0.9395 / 0.9768	0.8903 / 0.9352	0.9086 / 0.9565

These results confirm that the rewritten requirement specifications maintain the original meaning while introducing variation in wording and sentence structure. This validation step is essential to ensure that the augmented data is both diverse and technically accurate. Such consistency is necessary to support effective training of AI models on real-world industrial instructions.

## 9 Data Post-processing

After generating multiple versions of the requirement specifications using large language models (LLMs), a post-processing step was necessary to ensure the quality and consistency of the dataset. This phase aimed to remove duplicated content, correct potential inconsistencies, and prepare the data for annotation.

LLMs sometimes produce identical or nearly identical outputs, and in some cases, introduce small errors, unclear phrasing, or deviations from the original specification. To address this, we applied an automated filtering step to remove exact and near-duplicate texts. A Python script was used to compare all generated outputs and retain only unique formulations, helping preserve linguistic diversity while eliminating unnecessary repetition.

In addition to duplication, some generated specifications included errors or deviated from the required format. To resolve this, a manual review was conducted on a representative sample of the dataset. Three domain experts reviewed the selected entries to check that each requirement specification was grammatically correct, followed the intended structure, and accurately described the system behavior. Any texts containing unclear descriptions, factual errors, or formatting issues were either corrected or excluded.

To verify that the LLM-generated specifications remained semantically aligned with the original descriptions, we also performed a quantitative analysis using cosine similarity on sentence embeddings. Table 11 summarizes the similarity scores across the three systems and the overall dataset. The average similarity was 0.9275, with a minimum of 0.7105 and a maximum of 0.9924, indicating that the rewritten specifications preserved the original meaning while introducing useful variation.

After filtering and reviewing the generated texts, the dataset was clean and diverse but remained unstructured. Although each requirement specification described the full behavior of a manufacturing system, it did not explicitly indicate which terms referred to specific components. To make the data suitable for supervised learning, a final step was required to add structure through annotation. This involved identifying and labeling relevant terms so that AI models could learn to detect them automatically. The next section explains how this was done using Named Entity Recognition (NER) to assign labels to key elements within each specification.

## 10 Data Labeling

To make the dataset usable for supervised learning, each requirement specification must be structured in a way that allows machines to detect and classify key elements, such as the input and output components of a manufacturing system. Although the generated texts describe system behavior clearly, they do not explicitly indicate which words refer to physical components. To address this, we manually labeled the dataset using Named Entity Recognition (NER), following a consistent and interpretable annotation format.

**Fig. 7:**
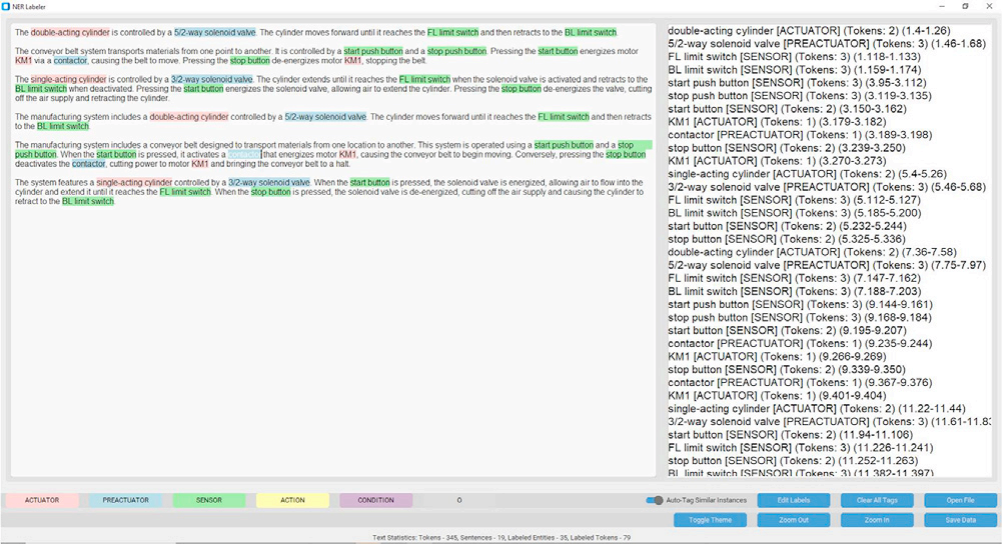
User Interface of Our Custom Labeling Tool.

Annotation was carried out by our research team, composed of three domain experts. The process was conducted entirely manually using our offline labeling tool, AutoLabel-NER, developed to support annotation of technical texts. As shown in Fig. 7, the tool provides a simple interface that allows annotators to assign a label to each token in a requirement specification. Each token is categorized into one of five NER classes: Sensor, Actuator, Pre-actuator, Effector, or Others. To ensure structural clarity, we used the standard BIO tagging scheme, where multi-word component names such as double acting cylinder are encoded as B-ACTUATOR, I-ACTUATOR, I-ACTUATOR. This format is widely used in NER applications to distinguish the beginning and continuation of entity spans.

The full dataset includes 2358 annotated requirement specifications. To ensure a manageable and high-quality review process, a subset of 300 specifications was selected for cross-verification. The dataset was equally divided among the three annotators. Each expert labeled approximately one-third of the total (around 786 specifications), and a rotational review procedure was implemented. Annotator 1 reviewed 100 examples from Annotator 2, Annotator 2 reviewed 100 from Annotator 3, and Annotator 3 reviewed 100 from Annotator 1. This process supported consistency checking across the team.

This review process helped identify disagreements and guided harmonization of annotation practices. Using this shared subset, Cohen’s Kappa was computed to measure inter-annotator agreement. The final average kappa score was 0.856, which indicates strong agreement.

The distribution of token-level annotations within the reviewed sample is provided in Table 12, while Table 13 presents the computed inter-annotator agreement scores.

**Table 12:** Token Counts per NER Label Across 300 Reviewed Requirement Specifications.

Label	Annotator 1	Annotator 2	Annotator 3	Total
O	7078	7162	6975	21,215
B-EFFECTOR	597	585	618	1800
I-EFFECTOR	492	485	539	1516
B-ACTUATOR	480	502	473	1455
I-ACTUATOR	343	369	355	1067
B-SENSOR	457	463	457	1377
I-SENSOR	630	633	618	1881
B-PREACTUATOR	116	136	116	368
I-PREACTUATOR	126	154	136	416
**Total Tokens**	**10,319**	**10,489**	**10,287**	**31,095**

**Table 13:** Inter-annotator agreement scores (Cohen’s Kappa).

Annotator Pair	Kappa Score
Annotator 1 & 2	0.856
Annotator 2 & 3	0.789
Annotator 1 & 3	0.924
**Average**	**0.856**

This manual annotation process transformed the dataset from unstructured text into a labeled resource suitable for training AI systems to identify and extract relevant entities (Table 14). The NER labels directly support the detection of input and output components in requirement specifications, which is essential for downstream applications in control logic synthesis and industrial automation.

**Table 14:** Sample of NER-Annotated Requirements with POS Tags and Tokenization.

Sentences	Tokens	Part of speech tags	NER labels
The system operates with two conveyor belts, named Conveyor One and Conveyor Two, each driven by an electric motor.	[‘The’, ‘system’, ‘operates’, ‘with’, ‘two’, ‘conveyor’, ‘belts’, ‘,’, ‘named’, ‘Conveyor’, ‘One’, ‘and’, ‘Conveyor’, ‘Two’, ‘,’, ‘each’, ‘driven’, ‘by’, ‘an’, ‘electric’, ‘motor’, ‘.’]	[‘DT’, ‘NN’, ‘VBZ’, ‘IN’, ‘CD’, ‘NN’, ‘NNS’, ‘,’, ‘VBN’, ‘NNP’, ‘CD’, ‘CC’, ‘NNP’, ‘CD’, ‘,’, ‘DT’, ‘VBN’, ‘IN’, ‘DT’, ‘JJ’, ‘NN’, ‘.’]	[‘O’, ‘O’, ‘O’, ‘O’, ‘B-EFFECTOR’, ‘I-EFFECTOR’, ‘I-EFFECTOR’, ‘O’, ‘O’, ‘B-EFFECTOR’, ‘I-EFFECTOR’, ‘O’, ‘B-EFFECTOR’, ‘I-EFFECTOR’, ‘O’, ‘O’, ‘O’, ‘O’, ‘O’, ‘B-ACTUATOR’, ‘I-ACTUATOR’, ‘O’]
The cycle is initiated by pressing the start button.	[‘The’, ‘cycle’, ‘is’, ‘initiated’, ‘by’, ‘pressing’, ‘the’, ‘start’, ‘button’, ‘.’]	[‘DT’, ‘NN’, ‘VBZ’, ‘VBN’, ‘IN’, ‘VBG’, ‘DT’, ‘NN’, ‘NN’, ‘.’]	[‘O’, ‘O’, ‘O’, ‘O’, ‘O’, ‘O’, ‘O’, ‘B-SENSOR’, ‘I-SENSOR’, ‘O’]
This causes the start light to come on and Conveyor One to begin moving.	[‘This’, ‘causes’, ‘the’, ‘start’, ‘light’, ‘to’, ‘come’, ‘on’, ‘and’, ‘Conveyor’, ‘One’, ‘to’, ‘begin’, ‘moving’, ‘.’]	[‘DT’, ‘VBZ’, ‘DT’, ‘NN’, ‘NN’, ‘TO’, ‘VB’, ‘IN’, ‘CC’, ‘NNP’, ‘NNP’, ‘TO’, ‘VB’, ‘VBG’, ‘.’]	[‘O’, ‘O’, ‘O’, ‘B-ACTUATOR’, ‘I-ACTUATOR’, ‘O’, ‘O’, ‘O’, ‘O’, ‘B-EFFECTOR’, ‘I-EFFECTOR’, ‘O’, ‘O’, ‘O’, ‘O’]
The box moves along Conveyor One until it is detected by Sensor A.	[‘The’, ‘box’, ‘moves’, ‘along’, ‘Conveyor’, ‘One’, ‘until’, ‘it’, ‘is’, ‘detected’, ‘by’, ‘Sensor’, ‘A’, ‘.’]	[‘DT’, ‘NN’, ‘VBZ’, ‘RB’, ‘NNP’, ‘CD’, ‘IN’, ‘PRP’, ‘VBZ’, ‘VBN’, ‘IN’, ‘NNP’, ‘NNP’, ‘.’]	[‘O’, ‘O’, ‘O’, ‘O’, ‘B-EFFECTOR’, ‘I-EFFECTOR’, ‘O’, ‘O’, ‘O’, ‘O’, ‘O’, ‘B-SENSOR’, ‘I-SENSOR’, ‘O’]
When Sensor A triggers, Conveyor One stops and Conveyor Two is started.	[‘When’, ‘Sensor’, ‘A’, ‘triggers’, ‘,’, ‘Conveyor’, ‘One’, ‘stops’, ‘and’, ‘Conveyor’, ‘Two’, ‘is’, ‘started’, ‘.’]	[‘WRB’, ‘NNP’, ‘NNP’, ‘NNS’, ‘,’, ‘NNP’, ‘NNP’, ‘NN’, ‘CC’, ‘NNP’, ‘CD’, ‘VBZ’, ‘VBN’, ‘.’]	[‘O’, ‘B-SENSOR’, ‘I-SENSOR’, ‘O’, ‘O’, ‘B-EFFECTOR’, ‘I-EFFECTOR’, ‘O’, ‘O’, ‘B-EFFECTOR’, ‘I-EFFECTOR’, ‘O’, ‘O’, ‘O’]

This initial version of the AutoFactory dataset represents a significant contribution to the field of AI for manufacturing. By releasing it to the research community, the objective is to accelerate progress in systems that can interpret natural language requirement specifications and convert them into executable control logic. The next stage involves fine-tuning large language models (LLMs) on this labeled data to evaluate their ability to extract components and understand system behavior from natural language.

### 10.1 Benchmarking and Model Fine-tuning

The transformer models selected for this study were chosen to provide a representative sample of architectures commonly used in sequence labeling tasks (Table 15). Three models were evaluated: BERT-Base, BERT-Large, and DistilBERT-Base. These models differ in size, depth, and computational requirements, allowing for a comparative analysis of performance versus efficiency on domain-specific Named Entity Recognition (NER) tasks.

**Table 15:** Architectural Comparison of the Transformer Models.

Model	Configuration	Parameters	Layers	Hidden Size	Attention Heads	Developed By
BERT	Base	110M	12	768	12	Google AI
	Large	340M	24	1024	16	
DistilBERT	Base	66M	6	768	12	Hugging Face

BERT-Base includes 12 transformer layers, each with 768 hidden units and 12 self-attention heads, amounting to 110 million parameters. BERT-Large expands this architecture to 24 layers with 1024 hidden units and 16 attention heads, resulting in a total of 340 million parameters. In contrast, DistilBERT-Base is a lighter model designed for faster training and inference. It compresses BERT-Base to 6 layers while retaining the same hidden size and number of attention heads, yielding a total of 66 million parameters.

These differences provide useful contrasts when assessing model suitability for industrial language processing, especially in scenarios where deployment constraints or training resources are limited.

To enable automatic identification of control components from natural language requirement specifications, the selected transformer models were fine-tuned on the annotated AutoFactory dataset using a Named Entity Recognition (NER) framework. The task involved classifying each token into one of five predefined categories: Actuator, Pre-actuator, Sensor, Effector, or Others, using the BIO tagging scheme. Fine-tuning was conducted on a consistent experimental setup to allow fair comparison.

All models were trained for three epochs with a learning rate of 5e-5 and a batch size of 16. The optimization objective was to minimize the cross-entropy loss between the predicted and true token-level labels. Evaluation was performed on a held-out test set representing 10 % of the dataset. Metrics used for performance assessment included Precision, Recall, F1 Score, and Evaluation Loss. These metrics were selected to reflect both the correctness and completeness of entity detection, which are critical for downstream automation tasks.

**Fig. 8:**
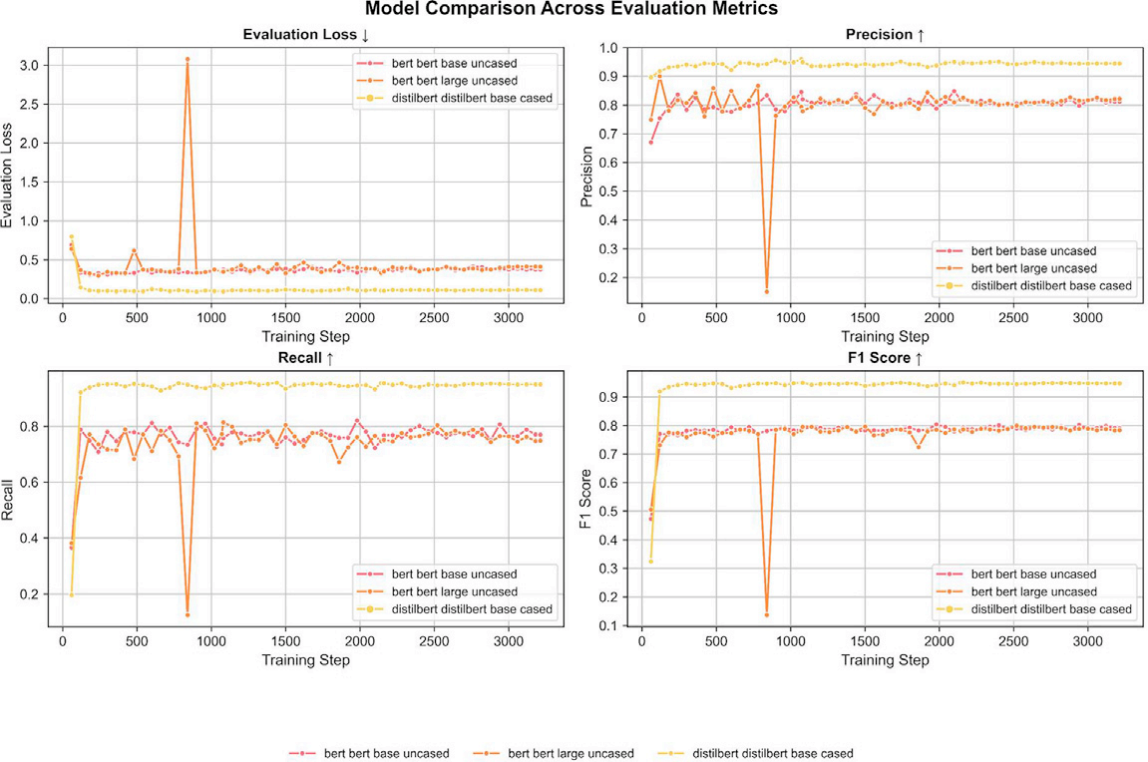
Model Performance Comparison Across Evaluation Metrics.

**Fig. 9:**
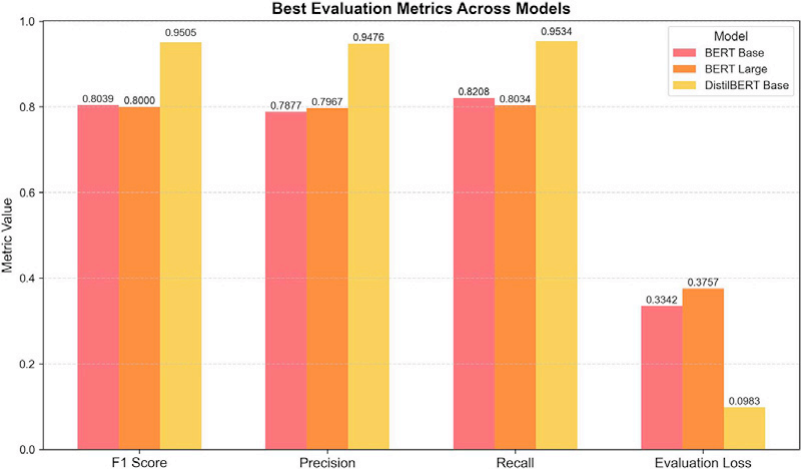
Evaluation of Transformer Models Based on Best F1-Scores.

The results clearly demonstrate that DistilBERT-Base achieved the highest performance across all evaluation metrics, with an F1 score of 0.9505, precision of 0.9476, and recall of 0.9534. Despite being the smallest model in terms of parameters, it outperformed the larger BERT models not only in accuracy but also in training stability and convergence. These findings are visually summarized in Fig. 8, Fig. 9 and Table 16.

**Table 16:** Performance Comparison of Transformer Models Fine-Tuned on the AutoFactory Dataset.

Model	Epochs	Learning Rate	Batch Size	Eval Loss	F1	Precision	Recall	Training Time (s)	Duration
BERT-Base	3	5,00E-05	16	0.3342	0.8039	0.7877	0.8208	1840.37	00:30:40
BERT-Large	3	5,00E-05	16	0.3757	0.8000	0.7967	0.8034	2857.11	00:47:37
DistilBERT-Base	3	5,00E-05	16	**0.0983**	**0.9505**	**0.9476**	**0.9534**	**1804.41**	**00:30:04**

The results confirm that the AutoFactory dataset is well-suited for training transformer models to recognize key control components in industrial requirement specifications. The fine-tuned models achieved high accuracy and demonstrated the potential for integrating such approaches into intelligent automation workflows.

## Limitations

The current version of the AutoFactory dataset includes a limited number of requirement specifications, covering approximately 3 out of 21 available scenarios from Factory I/O. While it addresses a range of manufacturing systems, the diversity of scenarios and components could be significantly expanded. Future updates will aim to include a broader variety of manufacturing systems and more comprehensive coverage of industrial processes. The dataset is currently available in CSV and XLSX formats. To enhance accessibility and usability, future versions will include additional file formats, such as JSON and XML, to accommodate different user preferences and requirements. All updates will be published on the same Hugging Face page mentioned in the “Data Accessibility” section of the table above. These updates will help the dataset grow and better support AI research in manufacturing.

## Ethics Statement

The authors have read and adhere to the ethical requirements for publication in Data in Brief. This work does not involve human subjects, animal experiments, or any data collected from social media platforms. It focuses solely on the creation and use of a dataset derived from synthetic requirement specifications generated by large language models for manufacturing systems, which was then carefully reviewed and verified by domain experts, ensuring compliance with ethical guidelines applicable to artificial intelligence research and data publication.

## Data Availability

Hugging FaceAutoFactory (Original data).
